# Supplementary material for: Causal Relationship between Aging and Anorexia Nervosa: A White-Matter-Microstructure-Mediated Mendelian Randomization Analysis
Source: Biomedicines. 2024 Aug 16;12(8):1874. doi: 10.3390/biomedicines12081874 (PMC11351342; doi:10.3390/biomedicines12081874)
Supplement: Supplementary file 1 [file biomedicines-12-01874-s001.zip › Supplementary Figure/Figure S8/Leaveoneout plot of MR analysis for TL to ubm-b-2022.pdf]

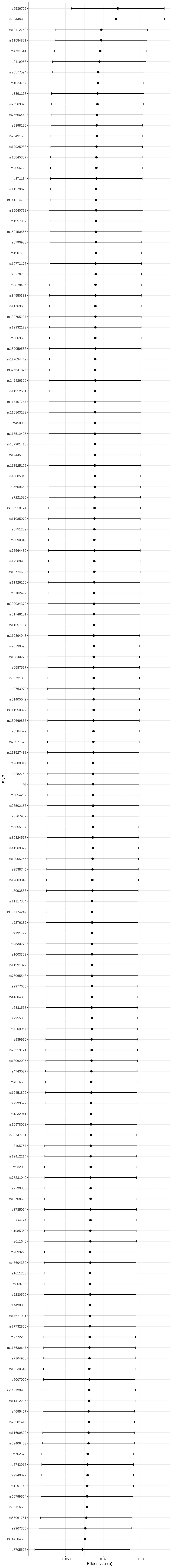
SNP
rs6536702
rs35446936
rs10112752
rs11584821
rs4731541
rs9419958
rs28577594
rs1023767
rs3891167
rs28363070
rs76666449
rs9398196
rs78491606
rs12925933
rs10845387
rs2056726
rs871134
rs11579626
rs141214782
rs35640778
rs1957937
rs150150565
rs6790988
rs1907702
rs10773176
rs6776756
rs9878436
rs34550383
rs11769630
rs139795227
rs12932179
rs6669563
rs182059586
rs117034449
rs376641875
rs142426306
rs11212631
rs117407747
rs116863223
rs450962
rs117512405
rs137901416
rs17445108
rs113525195
rs10805346
rs6659669
rs7221585
rs188918174
rs11085072
rs6751209
rs6590343
rs75664430
rs12369950
rs10774624
rs11428156
rs8102497
rs202034370
rs61748181
rs11557154
rs112394943
rs73730598
rs10840270
rs6587577
rs66731853
rs2763979
rs61405042
rs111950327
rs139669835
rs6584579
rs79977579
rs111527438
rs9600019
rs2282764
All
rs6054257
rs28502153
rs3767952
rs2555104
rs80324517
rs41269079
rs10905255
rs2538745
rs17803849
rs3093888
rs11117354
rs185174247
rs2276182
rs131797
rs4530278
rs1003322
rs11991877
rs76065543
rs2977608
rs41304632
rs6881568
rs9955360
rs7209057
rs939916
rs76219171
rs13062095
rs4743037
rs4616688
rs12451892
rs2293579
rs1332941
rs16978028
rs55747751
rs8105767
rs12412214
rs932002
rs77231040
rs7790856
rs10768683
rs3785074
rs4724
rs1985369
rs611646
rs7099229
rs45604339
rs1611236
rs869785
rs2230590
rs4498805
rs17677991
rs77732866
rs7772289
rs117630647
rs7164950
rs13230646
rs6007020
rs143190905
rs11412296
rs4695407
rs73581419
rs11699829
rs59409453
rs762679
rs5742915
rs9940099
rs1291143
rs56799554
rs80116508
rs56061761
rs2967355
rs144204502
rs7705526
-0.050
-0.025
0.000
Effect size (b)
